# State-of-the-Art Nanomaterials for Solar Cells

**DOI:** 10.3390/nano15070508

**Published:** 2025-03-28

**Authors:** Maykel Courel

**Affiliations:** Centro Universitario de los Valles (CUValles), Universidad de Guadalajara, Ameca 46600, Jalisco, Mexico; maykel.courel@academicos.udg.mx

The application of nanomaterials into solar cells has attained more and more attention from the scientific community in recent years. The fine control of nanomaterial properties such as size, shape, composition, etc., results in the tailoring of the material electrical and optical properties, which could be translated into an improved device performance. In other words, by changing the nanomaterial properties, it could be possible to achieve a higher photon absorption and thereby higher efficiencies. In this sense, experimental and theoretical research on the application of nanostructures to solar cells is needed. In particular, numerical and analytical simulations are valuable tools since they allow the optimization of both time and the cost of experimental processes. In addition, they favor the interpretation of developed measurements in complex structures, the design and optimization of solar cells, and the prediction of the optimal parameters that contribute to manufacturing a device with the best performance. Therefore, experimental works should be accompanied by theoretical studies to achieve a better understanding of devices. In this context, the Special Issue entitled “State-of-the-Art Nanomaterials for Solar Cells” is focused on experimental and theoretical studies on the application of nanomaterials to solar cells. This Special Issue brings together five articles, four research papers, and one review paper, dedicated to the application of nanomaterials to solar cells. Different topics concerning solar cells based on materials such as CdTe, CIGS, Kesterite, and Perovskites were analyzed. In particular, original results on micro-concentrator photovoltaics [1], the application of Cu_2_ZnSn(S,Se)_4_ quantum wells into Kesterite Solar Cells [2], the incorporation of ZnO:Al and CuSCN nanolayers on CdTe solar cells [3], and the proposal of electron selective layer-free perovskite solar cells [4], along with a review paper focused on the degradation of Perovskite solar cells [5] were presented. A short description of the main achievements is presented below:

Marina Alves et al. have discussed micro-concentrator photovoltaics (micro-CPVs) [1]. Micro-CPVs are an interesting proposal to reduce material usage and series resistance losses, resulting in the enhancement of solar cell conversion efficiency along with cost reduction. Particularly, CIGS micro-CPVs, which can be fabricated using either a top-down or bottom-up approach, have attracted increasing attention from the scientific community. The authors discussed the potential use of a bottom-up approach for CIGS micro-CPVs. For this purpose, pre-structured substrates were developed through photolithography, allowing easy modifications to the pattern design for different structures and geometries. CIGS micro-absorbers were prepared via sputtering of the CIG precursor into a patterned SiO_x_ matrix, followed by lift-off, thermal annealing at 500 °C, and a two-stage selenization process. The results indicated that both the as-deposited and annealed CIG films were Cu-poor, with CGI ratios of 0.88 and 0.84, respectively. The surface of the as-deposited CIG film exhibited roughness, characterized by CIG grains throughout the film. Subsequent thermal annealing at 500 °C led to less pronounced grains. In this work, it is further recommended to improve the CIGS absorber material by optimizing the two-stage selenization temperature and to produce complete CIGS microcells by deposing buffer and window layers.

Karina G. Rodriguez-Osorio et al. studied the potential use of Cu_2_ZnSn(S,Se)_4_ quantum wells in Kesterite solar cells [2]. This is a theoretical study aimed at enhancing device efficiency promotion through the incorporation of nanostructures. The physical mechanisms governing the optoelectronic parameters were analyzed with particular interest in the role of different well thickness, number, and anion composition. While efficiencies lower than 30% are demonstrated for bulk devices without nanostructures, the incorporation of quantum wells shows a significant short-circuit current-density improvement because of higher photon absorption, resulting in solar cell efficiencies that overcome the Shockley–Queisser limit for single junction solar cells. It was demonstrated that well thickness plays a more important role than well number, finding that the use of wells with thicknesses higher than 20 nm allows for better efficiency than those obtained for a device without nanostructures. A record efficiency of 37.5% is achieved when 36 wells with a width of 50 nm are used, considering an S/(S + Se) well compositional ratio of 0.25.

Isaac Montoya De Los Santos et al., in other theoretical work, evaluated the incorporation of ZnO:Al and CuSCN nanolayers on CdTe solar cell performance [3]. The influence of absorber and buffer thickness, absorber defect density, work function in back contact, series and shunt resistances, and carrier concentration on CdTe/CdS solar cell was studied to maximize its performance. An efficiency enhancement from 16.04% to 22.62% by increasing the J_sc_ and V_oc_ was reported with the incorporation of the nanolayers. This new proposal could guide the feasible fabrication of higher-efficiency CdTe-based photovoltaic cells.

Sajid Sajid et al. studied electron selective layers (ESLs)-free in perovskite solar cells (PSCs) [4]. ESLs-free PSC proposal is attractive since it allows the simplification of device layout, particularly avoiding the complex fabrication steps and multiple high-temperature treatment requirements. Nevertheless, this proposal results in the poor interface and inadequate quality of solution-processed perovskite thin films, inducing inefficient interfacial-charge extraction, thereby limiting the efficiency of ESL-free PSCs. By inserting an interfacial monolayer of diethanolamine (DEA) molecules between the perovskite and ITO substrate, a highly compact and homogenous perovskite thin film with large grains was reported. In addition, the DEA created a favorable dipole layer at the interface of perovskite and ITO substrate by molecular adsorption, which suppressed charge recombination. Solar cells based on DEA-treated ITO substrates resulted in efficiencies of up to 20.77%, which are one of the highest among ESL-free PSCs. It was also found that this technique successfully elongates the lifespan of ESL-free PSCs as 80% of the initial PCE was maintained after 550 h under AM 1.5 G irradiation at ambient temperature. This pivotal work opens up scientific pathways for the facile fabrication of ESL-free PSCs.

Bingchen et al. presented a review focused on achieving a better understanding of degradation details, including structural, compositional, morphological, and other changes in perovskite solar cells by utilizing in situ and operando approaches [5]. The review explored why these two approaches are necessary in the study of perovskite degradation and how they can be achieved by upgrading the corresponding ex situ techniques. With recent stability improvements of halide perovskite using various methods (compositional engineering, surface engineering, and structural engineering), the degradation of halide perovskite materials is greatly hindered. However, these improvements may turn into new challenges during the investigation into the hindered degradation process. Therefore, the authors also highlighted the importance of enhancing the sensitivity and probing range of current in situ and operando approaches to address this issue. Finally, the challenges and future directions of in situ and operando approaches in the stability research of halide perovskites were identified. It was pointed out that the advancement in in situ and operando techniques could be crucial in supporting the journey toward enhanced perovskite stability.

In short, the application of nanomaterials into solar cells is a promising area for achieving further solar cell efficiency promotion. Theoretical works have shown efficiencies higher than 30%. Therefore, further experimental and theoretical studies on the application of nanostructures into traditional solar cells based on CdTe, CIGS, Kesterite, and Perovskites are open research. This Special Issue presents the future path towards solar cell efficiency enhancement through the application of nanomaterials. Interesting proposals for efficiency enhancement in the different technologies are presented and discussed.
